# In Vitro Anti-Inflammatory and Vasculoprotective Effects of Red Cell Extract from the Black Sea Urchin *Arbacia lixula*

**DOI:** 10.3390/nu15071672

**Published:** 2023-03-29

**Authors:** Stefano Quarta, Egeria Scoditti, Vincenzo Zonno, Luisa Siculella, Fabrizio Damiano, Maria Annunziata Carluccio, Patrizia Pagliara

**Affiliations:** 1 Department of Biological and Environmental Sciences and Technologies (DISTEBA), University of Salento, 73100 Lecce, Italy; stefano.quarta3@unisalento.it (S.Q.); vincenzo.zonno@unisalento.it (V.Z.); luisa.siculella@unisalento.it (L.S.); fabrizio.damiano@unisalento.it (F.D.); 2 Institute of Clinical Physiology (IFC), National Research Council (CNR), 73100 Lecce, Italy; maria.carluccio@ifc.cnr.it

**Keywords:** sea urchin, red cells, endothelial dysfunction, inflammation, monocyte adhesion, atherosclerosis, cytokine, chemokine, adhesion molecule, NF-κB, gene expression

## Abstract

Sea urchins have emerged as an important source of bioactive compounds with anti-inflammatory and antioxidant properties relevant to human health. Since inflammation is a crucial pathogenic process in the development and progression of atherosclerosis, we here assessed the potential anti-inflammatory and vasculoprotective effects of coelomic red-cell methanolic extract of the black sea urchin *Arbacia lixula* in an in vitro model of endothelial cell dysfunction. Human microvascular endothelial cells (HMEC-1) were pretreated with *A. lixula* red-cell extract (10 and 100 μg/mL) before exposure to the pro-inflammatory cytokine tumor necrosis factor (TNF)-α. The extract was non-toxic after 24 h cell treatment and was characterized by antioxidant power and phenol content. The TNF-α-stimulated expression of adhesion molecules (VCAM-1, ICAM-1) and cytokines/chemokines (MCP-1, CCL-5, IL-6, IL-8, M-CSF) was significantly attenuated by *A. lixula* red-cell extract. This was functionally accompanied by a reduction in monocyte adhesion and chemotaxis towards activated endothelial cells. At the molecular level, the tested extract significantly counteracted the TNF-α-stimulated activation of the pro-inflammatory transcription factor NF-κB. These results provide evidence of potential anti-atherosclerotic properties of *A. lixula* red-cell extract, and open avenues in the discovery and development of dietary supplements and/or drugs for the prevention or treatment of cardiovascular diseases.

## 1. Introduction

Recently, research interest has increased in investigating natural products for their potential nutraceutical/pharmacological applications. Marine invertebrates, including echinoderms, are interesting sources of compounds, displaying biological activities [1,2,3]. Echinoderms play a key role in food webs and are a valuable resource for fisheries and aquaculture. Since ancient history, traditional Chinese medicine has used some species of echinoderms, especially sea urchins and sea cucumbers, to treat various diseases. More recently, many species belonging to this phylum have been found to contain promising bioactive compounds relevant to human health [3]. Indeed, antioxidant, anti-inflammatory, antifungal, antibacterial, antidiabetic, gastroprotective, anticardiotoxic and other bioactivities have been found in aqueous and organic extracts derived from spines, shells, gonads, intestines and coelomic fluid of different sea urchin species [4].

A sea urchin has a globose body, formed by a rigid test, with pedicellariae and mobile spines. The internal cavity hosts organs and a coelomic fluid that contains circulating cells called coelomocytes, which perform key roles including metabolite transport and immunity [5]. Various coelomocyte types can be recognized: phagocytes, vibratile cells, colorless spherule cells and red spherule cells [6]. These last cells accumulate around injuries and sites of infection, suggesting a role in the immune response. Red cells contain echinochrome A, a naphthoquinone responsible for their characteristic red color [7]. Echinochrome A shows similarities to histamine, displays antibacterial effects against both Gram-positive and Gram-negative bacteria, and triggers the inflammatory response as a part of the immune response to foreign pathogens [8]. Other polyhydroxylated naphthoquinone compounds, including spinochromes with immune-regulating and antioxidant properties, have been found in different parts of the sea urchin, including in red spherule cells [9,10]. These last cells have therefore been suggested as a potential source of therapeutic drugs/supplements with antioxidant and anti-inflammatory properties which are relevant to the prevention and/or treatment of human chronic degenerative diseases.

Cardiovascular disease is the leading cause of morbidity and mortality worldwide [11]. Oxidative stress and chronic low-grade inflammation are pathogenic determinants of the development and progression of atherosclerosis, the leading cause of cardiovascular disease [12]. The endothelium is crucial for vascular homeostasis, exerting several vasculoprotective effects, such as vasodilation, the inhibition of inflammatory responses, smooth muscle cell growth, and thrombosis [13]. During atherogenesis, upon exposure to pro-oxidant and pro-inflammatory stimuli such as smoking, hypertension, hyperglycaemia, or dyslipidaemia, endothelial cells become dysfunctional (i.e., activated), thus predisposing the patient to atherosclerotic plaque formation. Indeed, the production of vasodilating factors including nitric oxide and the endothelial barrier function is impaired [13]. In contrast, the expression of leukocyte adhesion molecules as well as the release of pro-thrombotic factors and pro-inflammatory cytokines and chemoattractants increase, in concomitance with the overproduction of reactive oxygen species (ROS) [13]. As a result, circulating monocytes are selectively recruited from the blood to the tunica intima, and differentiate into macrophages and, after taking-up lipids, into foam cells, which are characteristic of early atherogenesis. These events in concert further amplify the vascular inflammatory reaction, which characterizes and drives atherosclerosis progression and its clinical complications [14]. Scientific efforts have been dedicated to the discovery and clinical application of new, safe and effective drugs and/or supplements in the cardiovascular setting, and marine-sourced compounds may represent a valuable and mostly unexplored reserve [15].

In this context, experimental and clinical studies have provided evidence of the cardiovascular protective properties of sea urchin bioactive compounds, including red-cell-derived echinochrome A, which have been shown to prevent lipid peroxidation, platelet aggregation, cardiac and mitochondrial anomalies, vasoconstriction, and chronic low-grade inflammation [8,16,17,18]. Echinochrome A is the active ingredient of the histochrome drug, which is clinically used as an antioxidant in cardiology and ophthalmology [8]. However, whether part of the vasculoprotective effects of sea urchin extracts is mediated by an improvement of endothelial dysfunction is currently unknown.

Here, we aimed to assess the ability of coelomic red-cell extracts of the black sea urchin *Arbacia lixula* to prevent endothelial cell activation and monocyte recruitment, which are pathogenic determinants and therapeutic targets of atherosclerosis. For this purpose, an in vitro model of atherogenesis represented by vascular endothelial cells exposed to pro-inflammatory stimuli (i.e., TNF-α) [19] was used. A preliminary evaluation of the total phenolic content and antioxidant activity of the extract was also performed to verify the presence of compounds with antioxidant activity.

## 2. Materials and Methods

### 2.1. Chemicals

Tumour necrosis factor (TNF)-α was obtained from Sigma-Aldrich (now under Merck, Darmstadt, Germany). Unless otherwise specified, all other reagents were purchased from Sigma-Aldrich.

### 2.2. Sea Urchin Sampling, Red Cell Isolation and Extraction

Specimens of *Arbacia lixula* (7–8 cm diameter) were collected in the coastal area of Porto Cesareo (Northern Ionian Sea, Apulia, Italy) by SCUBA diving, at a depth of 5–10 m. The sea urchin coelomic fluid and red cell were obtained as previously described [20]. Extraction was performed on freeze-dried red cell samples. Briefly, 250 mg of ground freeze-dried red0cell biomass was extracted with 2.5 mL of methanol for 30 min and followed by centrifugation (12,000× *g* for 15 min). The supernatant was evaporated under vacuum. The dried extracts were weighted, solubilized in dimethyl sulfoxide (DMSO), and stored at −20 °C until analysis. DMSO was pretested for cell toxicity and showed no effects in terms of cell morphology, cell number, protein content, MTT as well as cell inflammation.

### 2.3. Determination of the Total Phenolic Content

The total phenolic content of *A. lixula* methanolic extract was determined using the Folin–Ciocalteau colorimetric assay [21]. The extract was solubilized in DMSO at a concentration of 1 mg/mL and added to the diluted Folin–Ciocalteu reagent. After 5 min, a 7.5% solution of Na_2_CO_3_ was added and incubated for 2 h. Absorbance was measured at 725 nm using a spectrophotometer. Concentrations were determined from a calibration curve of gallic acid (GA). Results are expressed in mg GA equivalent (GAE) per g of raw material. All determinations were performed in triplicate.

### 2.4. Antioxidant Activity Assays

The scavenging activity of *A. lixula* methanolic extract was detected using the Trolox equivalent antioxidant capacity (TEAC) assay, as previously described [2]. Briefly, an *A. lixula* extract at a concentration of 1 mg/mL and different concentrations of standard (Trolox) were mixed 1:1 (*v*/*v*) with the 2,2′-azinobis-(3-ethylbenzothiazoline-6-sulfonate) (ABTS) working solution and incubated for 6 min. Absorbance was then read at 734 nm. The radical scavenging potential (activity) of ABTS in the extract (percent of inhibition) was measured from the standard curve. Activities were reported as Trolox equivalents (TE) per g of raw material. All determinations were performed in triplicate.

### 2.5. Cell Culture and Treatments

The human microvascular endothelial cell line (HMEC-1) provided by E.W. Ades (Centers for Disease Control, Atlanta, GA, USA) [22] was cultured in an MCDB-131 medium supplemented with 15% foetal bovine serum (FBS), as previously described [23]. THP-1 monocytic cells were obtained from the American Tissue Culture Collection (Rockville, MD, USA) and cultured as previously described [24]. For cell treatments, HMEC-1 (at confluence) were cultured in the absence or presence of *A. lixula* methanolic extract (10 and 100 μg dry weight/mL) for 4 h and then stimulated with 10 ng/mL TNF-α for 0–18 h.

### 2.6. Cell Viability

The 3(4,5dimethylthiazol2yl) 2,5diphenyltetrazolium bromide (MTT) assay was used to determine cell viability. Briefly, after treatment with *A. lixula* extract and TNF-α stimulation, cells were exposed to MTT (0.5 mg/mL) for 2 h. The formazan products were then dissolved by isopropanol, and absorbance was measured at 595 nm.

### 2.7. RNA Isolation and Real-Time Quantitative Polymerase Chain Reaction

Total RNA was isolated using the TRIzol reagent (Invitrogen, Carlsbad, CA, USA) and reverse transcribed into cDNA using the High Capacity cDNA Reverse Transcription Kit (Applied Biosystems, Monza, Italy). A real-time quantitative polymerase chain reaction (qPCR) was performed in a CFX Connect Real-Time PCR Detection System (Bio-Rad Laboratories, Milan, Italy). The comparative critical threshold (ΔΔCT) method was used to calculate the amounts of mRNA, which were normalized to the expression levels of the GAPDH gene as the endogenous control. Results are expressed as fold increase relative to unstimulated control (made = 1). The primer sequences used are listed in Table 1.

### 2.8. Cell Surface Immunoassay

Surface expression of cell adhesion molecules on HMEC-1 was measured by the surface enzyme immunoassay (EIA) using mouse monoclonal antibodies against ICAM-1 and VCAM-1, as previously described [23].

### 2.9. Leukocyte-Endothelial Adhesion Assay

THP-1 cells (10^6^ cells/mL) were added to the treated HMEC-1 monolayers. After 30 min incubation, nonadherent cells were removed and adherent THP-1 cells were visualized with a phase contrast microscope (10 × bjective) connected to a digital camera (EVOS XL Core Imaging System, Thermo Fisher Scientific, Waltham, MA, USA). Adherent monocytes were counted using the ImageJ program (http://imagej.nih.gov/ij/ (accessed on 10 January 2023)).

### 2.10. THP-1 Chemotaxis Assay

The migration of THP-1 cells towards media collected from treated HMEC-1 was studied in a Boyden chamber (purchased by Corning through Sigma Aldrich, St. Louis, MO, USA) as previously described [23], with the upper and lower chambers separated by a polycarbonate membrane (8 µm pore size).

### 2.11. Preparation of Nuclear Extracts and Measurement of NF-κB p65 DNA Binding Activity

HMEC-1 were pretreated with *A. lixula* extract for 4 h and stimulated with 10 ng/mL TNF-α for 1 h. Nuclear proteins were isolated using the Nuclear Extract kit (Active Motif, Carlsbad, CA, USA) according to the manufacturer’s protocol. The activation of NF-κB was assessed using the ELISA-based TransAM NF-κB p65 kit (Active Motif, Carlsbad, CA, USA) following the manufacturer’s protocol.

### 2.12. Statistical Analysis

Results are expressed as means ± SD of at least three independent experiments performed in triplicate. Student’s *t* test was used for comparison of means between the control group and compound-treated group. Multiple comparisons were performed by one-way analysis of variance (ANOVA) using Bonferroni’s multiple comparison test. A *p* level of <0.05 was considered to be statistically significant.

## 3. Results

### 3.1. Total Polyphenol Content and Radical Scavenging Activity of Arbacia lixula Extract

Three different samples of methanolic extract of *A. lixula* red cells were independently analyzed for their total phenolic content (TPC). After solubilization in DMSO at a concentration of 1 mg/mL, the TPC of each solution was determined by performing a Folin–Ciocalteau assay. The mean phenolic compounds content was 15 ± 2.4 mg GAE/g. Since phenolic compounds are endowed with anti-inflammatory and antioxidant activity [25], before evaluating the effects of *A. lixula* red-cell extract on the inflamed endothelium, we first investigated the antioxidant activity of the extract by performing the TEAC assay. The mean antioxidant activity of the extract was 11 ± 2.6 µmol TE/g.

### 3.2. Effect of Arbacia lixula Extract on Endothelial Cell Viability

**Figure 1:**
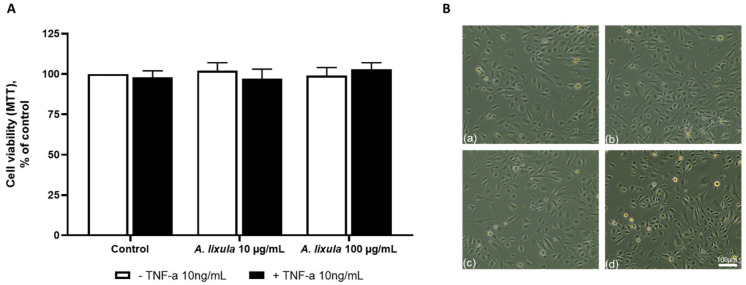
Effect of *A. lixula* extract on endothelial cell viability. HMEC-1 were treated with *A. lixula* extract for 4 h at the concentrations indicated, and then either treated with 10 ng/mL TNF-α or left untreated for 18 h. (**A**) Cell viability was assessed by the MTT assay and data (means ± S.D., *n* = 3) expressed as percent of unstimulated control. In (**B**), representative phase-contrast images (10× magnification) of cells after treatments are shown. (**a**) control; (**b**) TNF-α 10 ng/mL; (**c**) *A. lixula* extract 10 µg/mL + TNF-α; (**d**) *A. lixula* extract 100 µg/mL + TNF-α.

Preliminary experiments were conducted to evaluate the effects of *A. lixula* extract on endothelial cell viability in the presence or absence of TNF-α. HMEC-1 at confluence were pre-treated with *A. lixula* extract for 4 h; then they were stimulated with the cytokine TNF-α 10 ng/mL for 18 h. After cell treatments, viability was measured using the MTT assay, the total protein content, and cell morphology. As shown in Figure 1A, 10 ng/mL TNF-α alone did not influence cell viability. *A. lixula* extract, at any concentration tested, did not affect cell viability, either in the absence or in the presence of TNF-α. No change was seen in cell morphology under phase-contrast microscopy (Figure 1B). Therefore, we used 10 and 100 µg/mL as the concentrations of *A. lixula* extract for further experiments.

### 3.3. Arbacia lixula Extract Inhibits Monocyte Adhesion to Activated Endothelium

**Figure 2:**
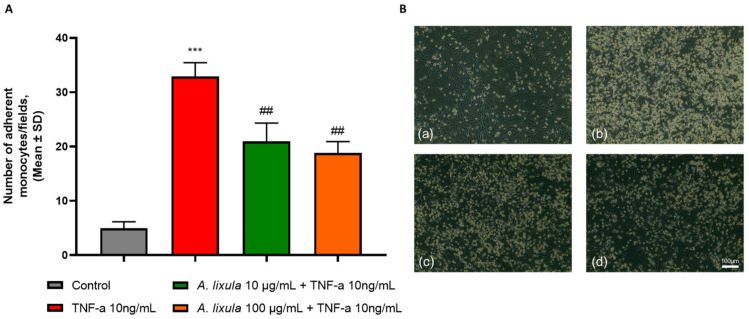
Effect of *A. lixula* extract on TNF-α-induced endothelial cell–monocyte adhesion. HMEC-1 were treated with *A. lixula* extract for 4 h at the concentration indicated, and then either treated with 10 ng/mL TNF-α or left untreated for 18 h. THP-1 were added to the HMEC-1 monolayers. Images of HMEC-1 and adherent THP-1 cells were visualized and counted (**A**). Data (means ± S.D., *n* = 3) are expressed as number of adherent monocytes per field. In (**B**), images captured with a phase contrast microscope (10× magnification) are shown. (**a**) control; (**b**) TNF-α 10 ng/mL; (**c**) *A. lixula* extract 10 µg/mL + TNF-α; (**d**) *A. lixula* extract 100 µg/mL + TNF-α. *** *p* < 0.001 vs. basal (untreated) control; ## *p* < 0.01 vs. TNF-α alone.

As shown in Figure 2, endothelial cells exposed to the cytokine TNF-α undergo proinflammatory changes that induce an increased adhesiveness of monocytes, being the prototypical sign of endothelial activation during atherogenesis. The pre-treatment of HMEC-1 with *A. lixula* extract before stimulation with TNF-α significantly reduced the number of monocytes that adhered to the endothelial cell monolayer compared with TNF-α alone.

### 3.4. Arbacia lixula Extract Decreases the Stimulated Expression of Adhesion Molecules

**Figure 3:**
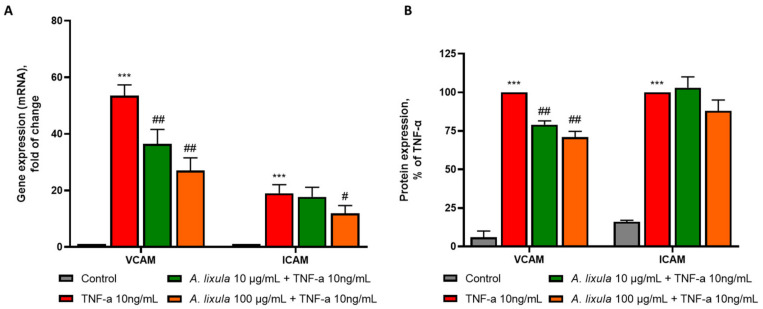
Effect of *A. lixula* extract on TNF-α-induced expression of endothelial adhesion molecules. HMEC-1 were treated with *A. lixula* extract for 4 h at the concentration indicated, and then either treated with 10 ng/mL TNF-α or left untreated for 18 h. (**A**) mRNA levels of VCAM-1 and ICAM-1 were measured by qPCR. Data (means ± S.D., *n* = 3) are expressed as fold induction over basal (untreated) control. *** *p* < 0.001 vs. basal (untreated) control; # *p* < 0.05 vs. TNF-α alone; ## *p* < 0.01 vs. TNF-α alone. (**B**) Endothelial cell surface protein expression of VCAM-1 and ICAM-1 was assessed by EIA and expressed as percent of TNF-α. *** *p* < 0.001 vs. basal (untreated) control; ## *p* < 0.01 vs. TNF-α alone.

Monocyte adhesion to the activated endothelium is mainly driven by de novo or increased expression of the adhesion molecules VCAM-1 and ICAM-1 on the vascular endothelial cells [26]. Therefore, we determined whether *A. lixula* extract affected the endothelial expression of these adhesion molecules. In accordance with the effect on monocyte adhesion, *A. lixula* extract reduced the stimulated mRNA (Figure 3A) and protein (Figure 3B) expression of VCAM-1 at both the concentrations tested. However, regarding ICAM-1, only the mRNA expression was downregulated by *A. lixula* extract (Figure 3A), and this occurred at the highest concentration tested (100 µg/mL). The ICAM-1 protein expression levels were reduced without reaching statistical significance (Figure 3B).

### 3.5. Arbacia lixula Extract Attenuates the Stimulated Expression of Inflammatory Genes and Monocyte Chemotaxis

**Figure 4:**
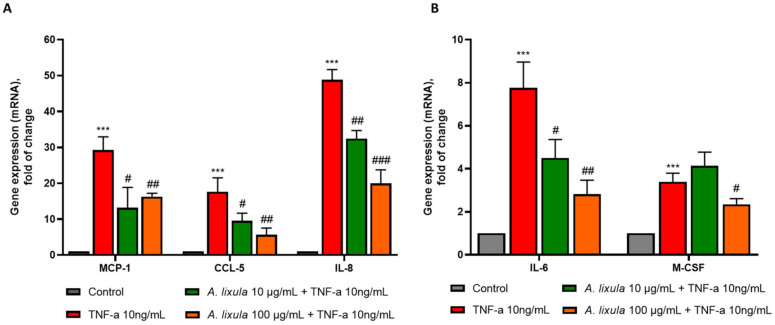
Effect of *A. lixula* extract on TNF-α-induced expression of inflammatory genes in human endothelial cells. HMEC-1 were treated with *A. lixula* extract for 4 h at the concentration indicated, and then either treated with 10 ng/mL TNF-α or left untreated for 18 h. mRNA levels of MCP-1, CCL-5, IL-8 (**A**), IL-6, and M-CSF (**B**) were measured by qPCR. Data (means ± S.D., *n* = 3) are expressed as fold induction over basal (untreated) control. *** *p* < 0.001 vs. basal (untreated) control; # *p* < 0.05 vs. TNF-α alone; ## *p* < 0.01 vs. TNF-α alone; ### *p* < 0.001 vs. TNF-α alone.

Endothelial activation and dysfunction are also characterized by an increased expression of inflammatory cytokines and chemokines that orchestrate the recruitment of monocytes to the vessel wall [14]. Among them, we tested the effects of *A. lixula* extract on the TNF-α-stimulated mRNA expression of chemokines, i.e., MCP-1 (CCL-2), CCL-5, IL-8, and cytokines also involved in monocyte maturation, differentiation and macrophage survival, i.e., IL-6 and M-CSF. Pre-treatment of HMEC-1 with *A. lixula* extract before TNF-α exposure significantly prevented MCP-1, CCL-5, IL-8 (Figure 4A), and IL-6 at both the concentrations tested, and M-CSF at 100 µg/mL (Figure 4B).

**Figure 5:**
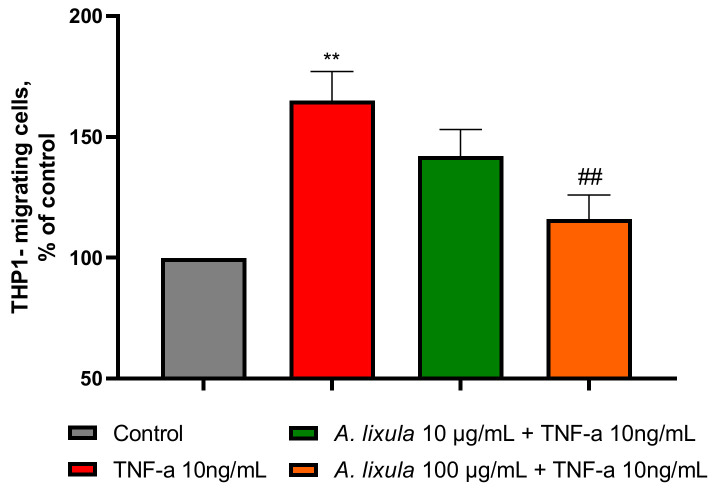
Effect of *A. lixula* extract on TNF-α-induced chemiotaxis of monocytes. HMEC-1 were treated with *A. lixula* extract for 4 h at the concentration indicated, and then either treated with 10 ng/mL TNF-α or left untreated for 18 h. Culture medium was collected and added to the lower chamber of a Boyden chamber. THP-1 were added to the upper chamber. Migrated THP-1 cells were then measured by the MTT assay. Data (means ± S.D., *n* = 3) are expressed as percent of untreated control. ** *p* < 0.01 vs. basal (untreated) control; ## *p* < 0.01 vs. TNF-α alone.

These inhibitory effects by *A. lixula* on pro-inflammatory gene expression in the endothelial cells agreed with the observation of a significant decreased monocytes chemotaxis towards conditioned media of HMEC-1 pretreated with *A. lixula* extract (100 µg/mL) compared with TNF-α alone (Figure 5).

### 3.6. Arbacia lixula Extract Decreases the Activation of NF-κB

**Figure 6:**
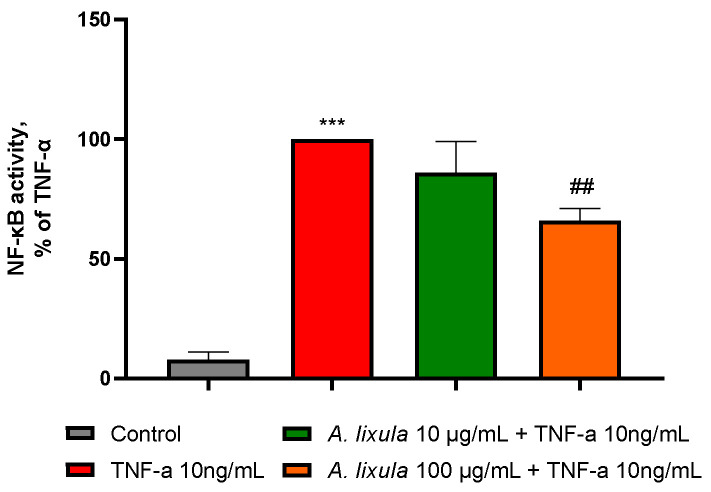
Effect of *A. lixula* extract on TNF-α-induced NF-κB activation. HMEC-1 were treated with *A. lixula* extract for 4 h at the concentration indicated, and then either treated with 10 ng/mL TNF-α or left untreated for 1 h. Nuclear proteins were analyzed for NF-κB p65 DNA-binding activity by ELISA. Data (means ± S.D., *n* = 3) are expressed as percent of TNF-α. *** *p* < 0.001 vs. basal (untreated) control; ## *p* < 0.01 vs. TNF-α alone.

Nuclear factor-κB (NF-κB) is a well-established transcription factor that plays a pivotal role in atherosclerosis by regulating the expression of adhesion molecules, cytokines, chemokines and other pro-inflammatory genes [27]. To elucidate the potential molecular mechanisms of *A. lixula* extract, we hypothesized that an effect on the NF-κB signaling pathway is activated by TNF-α. As shown in Figure 6, while TNF-α alone significantly enhanced the NF-κB p65 DNA binding activity, pretreatment with 100 µg/mL *A. lixula* extract meaningfully attenuated the stimulated DNA binding activity of NF-κB p65. This result suggests that *A. lixula* extract dampens the activation of the NF-κB signaling pathway induced by TNF-α.

## 4. Discussion

The identification of bioactive natural products as potential candidates for the development of safe and effective drugs and/or supplements for the treatment of chronic degenerative diseases is an active field of research. Work is particularly intense regarding cardiovascular diseases where, despite advancements in medical and surgical therapies, new cardiovascular drug discovery and development remain a major challenge internationally [28]. Marine organisms provide an immense and still unexplored source for the discovery of new compounds with pharmacological/nutraceutical properties [1]. In this context, sea urchins have been shown to contain various compounds that play a crucial role for organismal homeostasis. The antioxidant, anti-inflammatory, immunomodulating and anti-microbial properties of these compounds can be also relevant to human health [4].

In the present study, we demonstrated that the methanolic extract of *A. lixula* red cells exert vasculoprotective effects on human endothelial cells by inhibiting endothelial cell activation and the consequent monocyte adhesion induced by pro-inflammatory and proatherogenic stimuli such as TNF-α.

The endothelial monolayer is the interface between blood and the vessel wall where atheroma develops, and its alterations occur early during atherogenesis. Atherogenic risk factors and inflammatory mediators may impair the production of endogenous vasodilators, such as nitric oxide, by endothelial cells and trigger an endothelial pro-inflammatory phenotype. This involves the expression of adhesion molecules that bind circulating leukocytes, mainly monocytes, to the endothelial surface, and of chemotactic cytokines (chemokines) and growth factors that promote trans-endothelial migration of the bound leukocytes towards the intima, as well as their maturation and inflammatory activation [13]. The accumulation of monocytes and monocyte-derived macrophages in the intima contributes to chronic inflammation and atherosclerotic plaque inception and progression [14].

VCAM-1 and ICAM-1 are the main adhesion molecules that play a crucial role in the focal rolling and adhesion of monocytes to the activated endothelium. Both molecules are upregulated by atherosclerotic plaques [29,30], and their blockage using monoclonal antibodies or genetic deficiency significantly inhibits monocyte adhesion to endothelial cells and protectsagainst atherosclerotic plaque formation [31,32,33,34]. We found that *A. lixula* extracts, at non-toxic concentrations (10 and 100 μg/mL), significantly (*p* < 0.01) decreased the cytokine-induced gene and protein expression of VCAM-1 in endothelial cells. Moreover, *A. lixula* extract at 100 μg/mL significantly decreased the mRNA expression of ICAM-1. The protein levels of ICAM-1 on the surface of the endothelial cells were also reduced but in a statistically insignificant manner, suggesting that different concentrations and/or treatment time would be necessary to disclose any significant modulating effect on ICAM-1 protein expression. As a functional counterpart of adhesion molecule inhibition, *A. lixula* extract attenuated monocyte adhesion to activated endothelial cells, which represents the very early step in atherogenesis.

*A. lixula* extract also dampened the upregulation of other pro-inflammatory and pro-atherogenic mediators, including cytokines and chemokines (MCP-1, CCL-5, IL-8, IL-6, M-CSF) in endothelial cells exposed to TNF-α, thus demonstrating a general anti-inflammatory and atheroprotective effect in the vascular endothelium. MCP-1, CCL-5, and IL-8 are potent chemotactic factors for inflammatory cells, mostly monocytes, and exert various pro-inflammatory effects within the developing atheroma [35,36,37]. They are highly expressed in human atherosclerotic lesions [38,39,40,41]. The genetic or pharmacological inhibition of these chemokines or their receptors inhibited plaque formation and macrophage accumulation in atherosclerosis-prone animal models [42,43,44,45,46]. Some of these chemokines, including CCL-5 and IL-8, can also bind to the endothelial surface and mediate thr arrest of rolling leukocytes on activated endothelium during early atherogenesis [36,37].

M-CSF mediates monocyte maturation, differentiation and monocyte/macrophage proliferation and survival [47,48,49]. A genetic knockout of M-CSF or its receptor dramatically decreased atherosclerotic lesion sizes in animal models [50]. Interestingly, an endothelial cell-specific knockout of M-CSF reduced atherosclerotic lesions by about 30% [49].

IL-6 is a pleiotropic pro-inflammatory cytokine which is found at high levels in human atherosclerotic plaques [41] and in the blood of patients with coronary artery disease [51]. Activated endothelial cells are an important source of IL-6 [52], which has been shown to exert various pro-atherogenic roles including the stimulation of vascular smooth muscle cells growth [53], extracellular matrix remodelling [54], inflammatory cells recruitment and activation in the vessel wall [55], and pro-thrombotic effects [56]. Exogenous IL-6 in the apolipoprotein E-deficient (ApoE^−^/^−^) model of atherosclerosis increased the formation of atherosclerotic lesions [57], while the IL-6 receptor antibody downregulated inflammation and reduced atherosclerotic lesion sizes [58].

Notably, the targeting of pathways of inflammatory cytokines has been proposed as a potential therapeutic approach in the treatment of atherosclerotic disease [59,60]. Furthermore, some of the clinical benefits of drugs such as statins and aspirin in the treatment of cardiovascular disease may be explained by the inhibition of pro-inflammatory cytokine/chemokine pathways in vascular and inflammatory cells [37].

The observed inhibition of endothelial pro-inflammatory activation by *A. lixula* extract, as marked by reduced adhesion molecule and cytokine/chemokine expression and reduced monocyte transendothelial migration, may prove effective in the protection against the development of atherosclerosis. However, further experimental studies, mostly performed in animal models, are warranted to confirm and substantiate these in vitro data.

A potential regulation of NF-κB by the red-cell extract of *A. lixula* was also assessed. NF-κB represents the master transcription factor of inflammatory responses in vascular disease as well as in other chronic inflammatory diseases [27]. NF-κB is responsible for the concerted induction of several endothelial genes involved in endothelial activation and atherosclerosis, including adhesion molecules, pro-inflammatory cytokines, and chemotactic factors [61]. NF-κB belongs to a family of inducible transcription factors that, under basal conditions, is sequestered in the cytoplasm in an inactive state by the inhibitory subunit IκB. In response to different stimuli such as TNF-α, bacterial lipopolysaccharide (LPS), hyperglycemia, shear stress, oxidative stress, and hypoxia/reperfusion, IκB is degraded, and NF-κB rapidly activates and translocates into the nucleus to initiate the transcription of immune and inflammatory genes [61]. We found that endothelial cell treatment with *A. lixula* extract significantly prevented the activation of NF-κB in response to TNF-α, thus modulating the pro-inflammatory response at a pre-transcriptional level. Targeting the NF-κB signaling pathway is a promising approach in the administration of anti-inflammatory therapies [61]. Indeed, several drugs, natural products and bioactive foods exert their anti-inflammatory properties via NF-κB inhibition [62,63,64].

Previous studies in different cell and animal models ascribed significant anti-inflammatory effects to sea urchin-derived compounds or crude extracts [3,4]. In agreement with our results, ovothiol A, having been isolated from the sea urchin eggs, inhibited the stimulated expression of adhesion molecules in endothelial cells and endothelial cell–monocyte adhesion [17]. Echinochrome A and other sea urchin pigments reduced the production of pro-inflammatory mediators (e.g., cytokines, metalloproteases and prostaglandins), the levels of ROS in inflammatory cells including macrophages, as well as tissue accumulation of inflammatory cells, and improved cardiomyocyte and cardiac progenitor cell function [3]. A portion of these effects was associated with the modulation of mitogen-activated protein kinase (MAPK) [65] and NF-κB pathways [66]. Other compounds which were isolated from sea urchins downregulated inflammatory responses, mainly those in immune cells, by inhibiting the MAPK and NF-κB pathways [67,68].

Here, we provided the first demonstration of anti-inflammatory vasculoprotective effects of *A. lixula* red-cell extract in endothelial cells via the inactivation of NF-κB, thus adding new evidence to the biological relevance and potential clinical utility of sea urchins to human health.

A portion of the beneficial effects of sea urchin constituents has been attributed to their antioxidant properties, as extensively demonstrated in cellular models and in vivo [3,4]. Indeed, oxidative stress is one of the basic pathogenic processes in several chronic diseases, including atherosclerosis, and is closely related to inflammation and endothelial dysfunction [69]. Oxidative stress can modify biomolecules, leading to tissue injury, and can activate several pro-inflammatory transcription factors, including NF-κB [69]. Therefore, the development of antioxidant treatments is an important therapeutic goal [70]. In using the TEAC assay, we found that the tested extract of *A. lixula* was endowed with antioxidant activity, confirming previous data on the same sea urchin species [2] and in agreement with findings on other sea urchin species and anatomical parts [71,72]. Although various compounds in sea urchins may contribute to the observed antioxidant activity, sea urchins have been shown to contain phenol components [71,73], i.e., secondary metabolites with potent antioxidant properties owing not only due to ROS scavenging and pro-oxidant metal ion chelation, but also due to the regulation of the cellular antioxidant systems [74]. Therefore, we measured the phenol content of *A. lixula* red-cell extract using the Folin–Ciocalteu assay and found the presence of phenol compounds at levels comparable or even higher than those found in other sea urchin species and anatomical parts [71,72,75], and comparable to those found in edible and non-edible plant extracts [76] (http://phenol-explorer.eu/food-processing/foods (accessed on 28 February 2023)).

These data underscore the potential utility of the tested extract as a significant source of natural antioxidants. Of course, further characterization of the phenolic composition of our extract is warranted. Moreover, based on its antioxidant content, the effects of *A. lixula* extract on intracellular ROS production should be verified. Pending further evaluations, we can speculate that the antioxidant (and phenol content) properties could contribute to the anti-inflammatory effects, shown here by the *A. lixula* red-cell extract in endothelial cells.

To our knowledge, the bioactivity of the whole coelomic red-cell extract has never been tested on markers of endothelial dysfunction. We here disclosed novel biological activities of sea urchin that may be derived from the presence of only partially identified compounds that might act in additive/synergistic interactions, mostly at the highest concentration tested, i.e., 100 μg/mL. As outlined above, a limitation of the present study is that the *A. lixula* coelomic red-cell extracts need to be further characterized in terms of the chemical constituents that may be responsible for the beneficial effects documented here.

In terms of ecological sustainability, *A. lixula* is a non-edible species and is not intensively harvested, as is presently carried out for other sea urchin species [77]. The natural populations may therefore still be abundant, at least in the coastal area of this study. To obtain higher quantities for industrial exploitation, *A. lixula* may be reproduced and cultivated on land, as has already been performed for other echinoderm species [78].

Overall, the results of the present study suggest that extracts of *A. lixula* red cells may be helpful in vascular protection against the development of early atherosclerosis, and open new potential perspectives onto the development of drugs/supplements for the prevention and/or treatment of cardiovascular disease.

## Figures and Tables

**Table 1:** Primer sequences.

Gene	Forward Primer	Reverse Primer
VCAM-1	5′-CATGGAATTCGAACCCAAAC-3′	5′-CCTGGCTCAAGCATGTCATA-3′
ICAM-1	5′-AGACATAGCCCCACCATGAG-3′	5′-CAAGGGTTGGGGTCAGTAGA-3′
MCP-1	5′-CCCCAGTCACCTGCTGTTAT-3′	5′-TCCTGAACCCACTTCTGCTT-3′
CCL-5	5′-CGCTGTCATCCTCATTGCTA-3′	5′-GAGCACTTGCCACTGGTGTA-3′
IL-8	5′-GTGCAGTTTTGCCAAGGAGT-3′	5′-CTCTGCACCCAGTTTTCCTT-3′
IL-6	5′-AGGAGACTTGCCTGGTGAAA-3′	5′-CAGGGGTGGTTATTGCATCT-3′
M-CSF	5′-TGGACGCACAGAACAGTCTC-3′	5′-CCTCCAGGGCTCACAATAAA-3′
GAPDH	5′-AAACGGCTACCACATCCAAG-3′	5′-CCTCCAATGGATCCTCGTTA-3′

VCAM-1: vascular cell adhesion molecule-1; ICAM-1: intercellular adhesion molecule-1; MCP-1: monocyte chemoattractant protein-1; CCL-5: chemokine (C-C motif) ligand-5; IL-8: interleukin-8; IL-6: interleukin-6; M-CSF: macrophage colony stimulating factor; GAPDH: glyceraldehyde-3-phosphate dehydrogenase.

## Data Availability

Data is contained within the article.
